# Automatic Brain Tumor Segmentation in 2D Intra-Operative Ultrasound Images Using Magnetic Resonance Imaging Tumor Annotations

**DOI:** 10.3390/jimaging11100365

**Published:** 2025-10-16

**Authors:** Mathilde Gajda Faanes, Ragnhild Holden Helland, Ole Solheim, Sébastien Muller, Ingerid Reinertsen

**Affiliations:** 1 Department of Health Research, SINTEF Digital, 7465 Trondheim, Norway; ragnhild.holden.helland@sintef.no (R.H.H.); sebastien.muller@sintef.no (S.M.);; 2 Department of Physics, Norwegian University of Science and Technology (NTNU), 7491 Trondheim, Norway; 3 Department of Circulation and Medical Imaging, Norwegian University of Science and Technology (NTNU), 7491 Trondheim, Norway; 4 Department of Neurosurgery, St. Olavs Hospital, 7030 Trondheim, Norway; 5 Department of Neuromedicine and Movement Science, Norwegian University of Science and Technology (NTNU), 7491 Trondheim, Norway

**Keywords:** brain tumors, deep learning, segmentation, ultrasound, intra-operative

## Abstract

Automatic segmentation of brain tumors in intra-operative ultrasound (iUS) images could facilitate localization of tumor tissue during the resection surgery. The lack of large annotated datasets limits the current models performances. In this paper, we investigated the use of tumor annotations in magnetic resonance imaging (MRI) scans, which are more accessible than annotations in iUS images, for training of deep learning models for iUS brain tumor segmentation. We used 180 annotated MRI scans with corresponding unannotated iUS images, and 29 annotated iUS images. Image registration was performed to transfer the MRI annotations to the corresponding iUS images before training the nnU-Net model with different configurations of the data and label origins. The results showed similar performance for a model trained with only MRI annotated tumors compared to models trained with only iUS annotations and both, and to expert annotations, indicating that MRI tumor annotations can be used as a substitute for iUS tumor annotations to train a deep learning model for automatic brain tumor segmentation in the iUS images. The best model obtained an average Dice score of 0.62 ± 0.31, compared to 0.67 ± 0.25 for an expert neurosurgeon, where the performance on larger tumors was similar, but lower for the models on smaller tumors. In addition, the results showed that removing smaller tumors from the training sets improved the results.

## 1. Introduction

Diffuse gliomas are primary brain tumors that infiltrate normal brain tissue, making complete surgical resection impossible [1]. Although incurable, treatment can prolong life and improve quality of life and brain functions [2]. Surgical resection is often the preferred primary treatment option, as the extent of tumor resection is linked to prolonged survival [3]. However, removing or damaging healthy surrounding tissue can negatively affect the patient function [4]. The precise tumor border localization is, therefore, crucial for successful resection.

Low-cost, real-time intra-operative ultrasound (iUS) has been shown to enhance the surgical outcome [5]. However, the images have a limited field of view and often contain noise and artifacts that make them difficult to interpret [6]. Automatic brain tumor segmentation in the iUS images could facilitate interpretation and help the surgeon perform a more complete resection.

Deep learning can address this segmentation task as demonstrated in the CuRIOUS-SEG challenge in 2022 [7]. The winning contribution came from Qayyum et al. [8] who achieved a Dice score of 0.57 using 23 annotated iUS images from RESECT [9] and RESECT-SEG [10] as the training set, which is to date the only publicly available annotated dataset. Recently, Dorent et al. [11] introduced a patient-specific segmentation model trained on simulated ultrasound images from pre-operative MRI scans from a single patient. Their approach obtained a median Dice score of 0.84–0.87, compared to 58.5–71.7 for a general model trained uing the RESECT dataset [11] as inputs. Despite good results, their approach is limited to pre-resection scenarios and requires time-consuming and computationally extensive training for each patient. Having a model that generalizes well to all patients would be more advantageous in the clinic, but the lack of large annotated iUS datasets limits further development.

To address this issue, we explored whether tumor annotations in pre-operative MRI scans could be used as a substitute for manual tumor delineations in the iUS images to expand the training set of a deep learning model for automatic brain tumor segmentation in the iUS images. MRI tumor annotations are more accessible than iUS tumor annotations, either from published datasets [12], or by software for automatic tumor segmentation such as Raidionics (v1.2) [13]. Since non-navigated 2D ultrasound imaging is more commonly used, and it is low cost than the 3D ultrasound imaging [5], this work focuses on 2D images. Training sets with only MRI tumor annotations, only iUS tumor annotations, and both MRI and iUS tumor annotation were used to train 2D deep learning models. All models were evaluated, using a separate test dataset with manual iUS annotations, and compared to the inter-observer variability.

## 2. Materials and Methods

**Figure 1:**
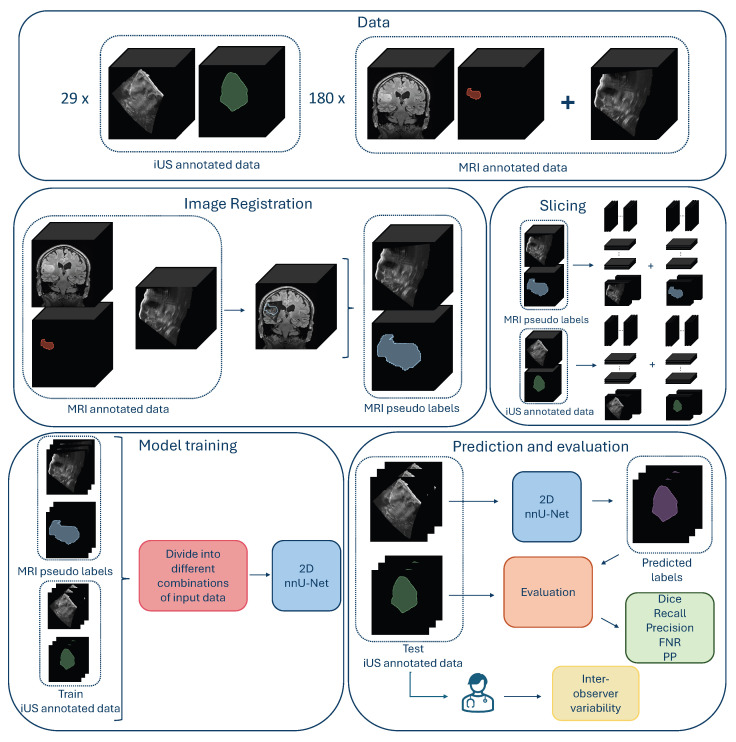
Overview over the data, data preparation, model training, and evaluation of the results. (The figure is best viewed in color in a digital version, which allows for zooming in).

An overview of the methods is shown in Figure 1.

### 2.1. Data

Both annotated and un-annotated iUS images were used in this study. Data were divided into two categories: iUS annotated data and MRI annotated data, as explained in Figure 1. The iUS annotated data consisted of iUS images with manual tumor annotations, whereas the MRI annotated data comprised iUS images without manual tumor annotations, but with pre-operative MRI scans with tumor annotations. Depending on the experiment, all or parts of the data were used for training and testing.

The iUS annotated data were obtained from 29 patients with gliomas, who underwent surgery at St. Olavs Hospital, Trondheim, Norway. Among these, 23 were published as part of the REtroSpective Evaluation of Cerebral Tumors (RESECT) dataset [9], where the annotations are available from the RESECT-SEG dataset [10]. The remaining 6 were used as the test set in the CuRIOUS-SEG challenge, organized in conjunction with the MICCAI 2022 conference [14]. The datasets contain the iUS images acquired before, during, and after resection. Only the images acquired before resection were used in this study, giving a total of 29 annotated 3D ultrasound images.

The MRI annotated data consisted of 180 images from the Brain Resection Multimodal Imaging Database (ReMIND) [12], and in-house data from St. Olavs University Hospital. From the ReMIND database, 103 iUS images acquired before resection with corresponding annotated pre-operative MRI scans from 55 patients with glioma or metastasis, who underwent surgery for the first time, were included in the study.

The in-house dataset from St. Olavs Hospital contained 77 pre-resection 3D iUS images with corresponding annotated pre-operative MRI scans from 43 patients with glioma or metastasis who underwent surgery for the first time. The ultrasound images were mainly acquired with a 12FLA-L linear probe, using the Sonowand Invite neuronavigation system [15] or the CustusX neuronavigation system [16]. Data were collected through the Central Norway Brain Tumor Registry and Biobank, or through several research projects on ultrasound-guided neurosurgery, with written informed consent from all patients [17]. For cases with missing tumor annotations, the open-source software for automatic brain tumor segmentation in MRI scans, Raidionics (v1.2) [13], was used to generate annotations. The software is thoroughly validated and has been used in various studies [18,19,20]. This resulted in 29 annotated 3D iUS images and 180 un-annotated 3D iUS images with corresponding pre-operative 3D MRI scans and annotations from 127 patients.

### 2.2. MRI–iUS Registration

To use the MRI tumor annotations as labels for the ultrasound images lacking annotations, the MRI tumor annotations were transferred to the corresponding ultrasound space by rigid registration using the medical image analysis software, ImFusion Suite (Version 2.42.2) [21]. The algorithm was proposed by Wein et al. [22], and has shown great performance on similar tasks [23,24]. All registrations were visually inspected to ensure adequate alignment.

### 2.3. Model Pre-Processing, Training, and Post-Processing

The nnU-Net framework was used in this study because it is a standardized and self-configuring deep learning framework, where optimal pre-processing, hyperparameters, and post-processing were determined based on the given dataset. In addition, it has shown state-of-the-art performance in many biomedical image segmentation tasks [25].

The iUS 3D volumes were sliced in all three perpendicular directions and saved in the NIfTI-format to obtain 2D images. Only tumor-containing slices were kept. The 2D configuration of nnU-Net was used with the nnUNetTrainerDA5 option. All models were trained with five-fold cross-validation with early stopping with a patience of 30 epochs, ensuring no information leaks by keeping all 2D slices from one patient in the same fold. The pre-processing, hyperparameters, and post-processing proposed by the framework for each dataset, were used.

An Intel Core Processor (Broadwell, no TSX, IBRS) CPU with 16 cores, 64 GB of RAM, Tesla V100S (32 GB) dedicated GPU, and a regular hard-drive were used for training. The nnU-Net framework v.2.2 was used with Python 3.8 and PyTorch v.2.2.0.

### 2.4. Experiments

#### 2.4.1. Comparison of Tumor Area Cut-Off Values

In this experiment, we used the iUS images with registered MRI labels from the MRI annotated data as explained in Figure 1. The 2D slices contained tumors with a vast variability in tumor areas. Slices from the edges of the tumor volumes were particularly small, and there was a higher risk of mismatch between the MRI pseudo label and the tumor in the iUS due to inaccurate image registration, compared to slices in the middle of the tumor volume. This experiment, therefore, studied different tumor area cut-off values, to investigate the effect of excluding small and possibly poorly aligned tumor labels that could be a source of noise rather than a valuable contribution to the training data. Nine models were trained in this experiment using 2D slices from the 180 3D MRI annotated images with tumor area cut-off values ranging from 0 to 300 mm^2^. The number of 2D slices used for training can be seen in Table 1. All models were evaluated for all tumor-containing slices of the 29 3D volumes of the iUS annotated data resulting in 14,107 test slices.

#### 2.4.2. Comparison Between MRI Labels, iUS Labels, and Manual Annotations

In this experiment, the best model from the first experiment was compared to a model trained using only iUS annotated data as the inputs, and a model trained using both iUS and MRI annotated data. For the iUS-model, the iUS annotated data were split into training and test sets. The 23 patients from RESECT were used for training, and the 6 patients from the test set of the CuRIOUS-SEG challenge were used as a test set, resulting in 2259 test 2D slices. For the MRI+US_200 model, eight 3D images from the MRI annotated data were excluded from the training set due to poor image quality. The number of 2D slices used for training is shown in Table 1. To ensure a fair comparison between the models, both models were trained using nnU-Net with a tumor area cut-off of 200 mm^2^ based on the results from the first experiment. The models are available here: https://github.com/mathildefaanes/us_brain_tumor_segmentation/tree/main (accessed on 1 October 2024).

**Table 1:** The Dice score (mean ± standard deviation) for the nine MRI annotated data models from Experiment 1 in the upper part of the table, and for the three models and the annotator from Experiment 2, in the lower part, evaluated on the test 2D slices from each experiment in different tumor area intervals; between 0–35 mm^2^, 35–200 mm^2^ and above 200 mm^2^, and on the total amount of test slices. For Experiment 1, * is showing statical significance from MRI_0 (*p* < 0.0064, Bonferroni) with effect sizes in parentheses. The percentage of positive predictions (PP) for the total test set and the number of training samples (N) written in thousands, are also shown. The arrows indicate which value is preferred, and bold values indicate the best scores in each column.

	Tumor Area Ranges [mm^2^]					
**Model**	**0–35 ↑**	**35–200 ↑**	**>200 ↑**	**Total ↑**	**PP ↑**	**N**
MRI_0	0.03 ± 0.10	0.20 ± 0.28	0.61 ± 0.30	0.45 ± 0.36 (-)	72.9%	105
MRI_5	0.05 ± 0.13	0.26 ± 0.30	0.63 ± 0.27	0.49 ± 0.34 * (0.07)	79.2%	100
MRI_15	0.06 ± 0.13	0.28 ± 0.29	0.64 ± 0.25	0.50 ± 0.33 * (0.10)	84.6%	95
MRI_25	0.05 ± 0.13	0.26 ± 0.30	0.63 ± 0.27	0.48 ± 0.34 * (0.07)	78.9%	92
MRI_35	0.07 ± 0.15	0.31 ± 0.30	0.68 ± 0.23	0.53 ± 0.32 * (0.18)	87.6%	89
MRI_70	0.06 ± 0.13	0.32 ± 0.30	0.67 ± 0.23	0.52 ± 0.32 * (0.16)	86.9%	80
MRI_100	0.07 ± 0.13	0.34 ± 0.29	0.70 ± 0.21	0.56 ± 0.31 * (0.23)	90.0%	73
MRI_200	**0.07** ± **0.09**	**0.40** ± **0.25**	0.75 ± 0.15	**0.60** ± **0.28** * **(0.25)**	98.6%	51
MRI_300	0.06 ± 0.07	0.38 ± 0.21	**0.76** ± **0.13**	0.60 ± 0.27 * (0.21)	**99.8**%	35
MRI_200	0.06 ± 0.09	0.33 ± 0.27	0.79 ± 0.15	0.58 ± 0.32	96.6%	51
MRI+US_200	0.08 ± 0.10	0.40 ± 0.27	**0.81** ± **0.12**	0.62 ± 0.31	99.0%	57
US_200	0.08 ± 0.10	0.37 ± 0.22	0.77 ± 0.12	0.59 ± 0.29	**100**%	8
Annotator	**0.20** ± **0.23**	**0.61** ± **0.26**	0.77 ± 0.14	**0.67** ± **0.25**	95.2%	-

In addition, the performance of the models was compared to the inter-observer variability, to assess the difficulty of the segmentation task. The first author manually annotated 3D images of the test set, using 3D Slicer (Version 5.2.2) [26]. The annotations were adjusted and validated by an experienced neurosurgeon (OS) and compared against the CuRIOUS-SEG ground truth annotations. These results, are presented in the following sections and referred to as “Annotator”.

### 2.5. Evaluation and Statistical Analysis

To evaluate the models in each experiment, the pixel-wise segmentation performance was evaluated on the test sets by calculating Dice score, precision, recall, and false negative rate (FNR) between the ground truth masks and the binary prediction masks. The percentage of positive predictions (PP), thus if one or more pixels were classified as a tumor in an image, was also calculated image-wise.

Linear regression models were used to assess statistical differences in Dice scores between the models in each experiment. The substantial within-patient correlation between 2D slices was considered by clustering on a patient level (Python package: pyfixest). To compensate for multiple pairwise comparisons between groups, the Bonferroni correction of type I error was applied. The effect size of the differences was given by Cohen’s $d=(\bar{x_{i}}-\bar{x_{j}})/s$, where $\bar{x_{i}}$ and $\bar{x_{j}}$ represent the average Dice score achieved with model *i* and *j*, and *s* is the pooled standard deviation calculated using clustered standard errors $S_{i}$ estimated by the regression [27]. As all groups had the same number of observations *n*, it reduces to $s^{2}=n\left(S_{i}^{2}+S_{j}^{2}\right)/2$. We used d∼0.01 describing very small and d∼0.2 describing small effect sizes [28].

In addition, the influence of the tumor area cut-off value in Experiment 1 was assessed using linear regression to predict Dice score, precision, recall, and FNR, correcting for within-patient correlation using the same method. A similar logistic regression was used for the binary positive prediction (PP).

## 3. Results

### 3.1. Comparison of Tumor Area Cut-Off Values

**Figure 2:**
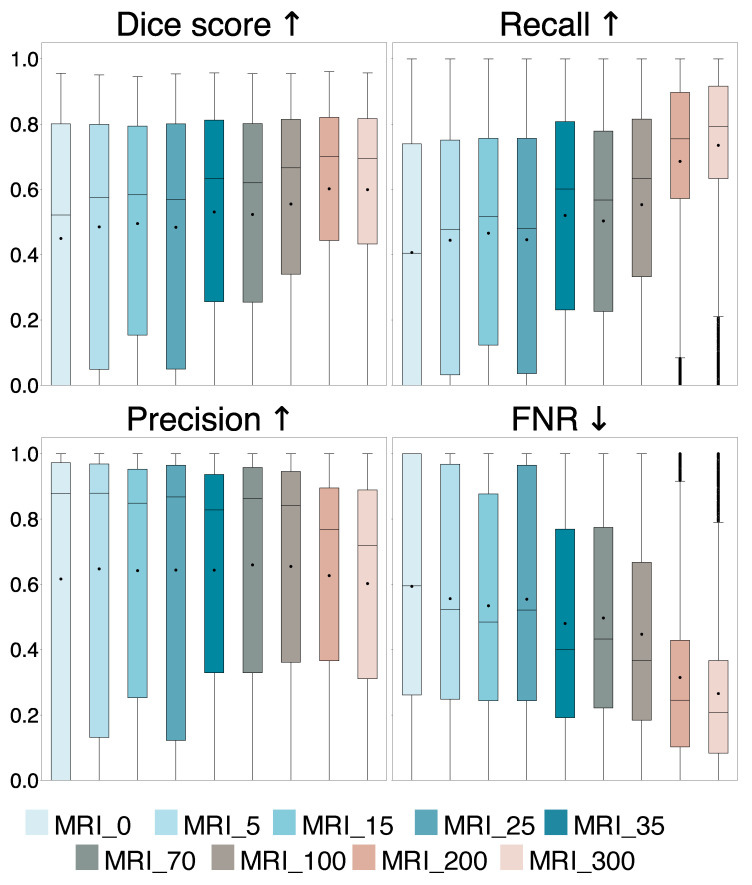
Comparison of Dice score, recall, precision, and false negative rate (FNR) for the nine models trained with different tumor area cut-off values, where MRI_0 is trained using all tumor-containing slices and MRI_5 to MRI_300 are trained with slices containing a tumor area larger than 5 mm^2^ up to 300 mm^2^, respectively. The average values are represented by dots and the median values are represented by lines. The arrows indicate which value is preferred.

**Figure 3:**
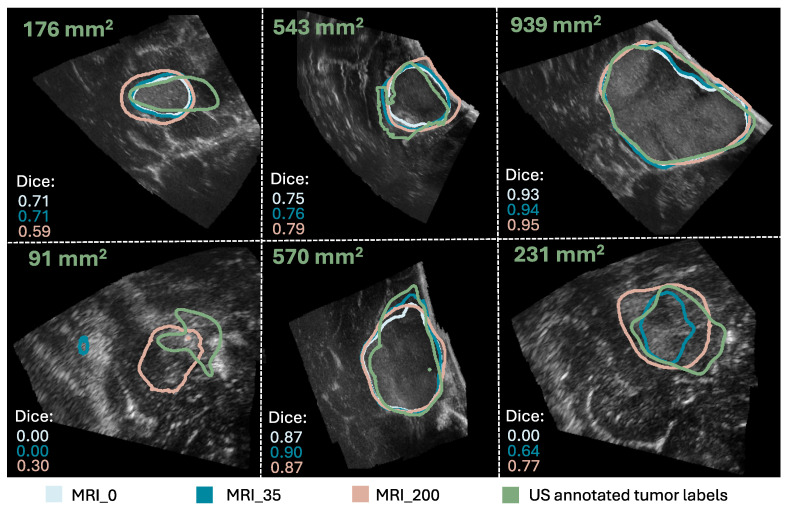
Comparison of the segmentation masks of the ground truth (in green), the MRI_0 model (in light blue), the MRI_35 model (in blue), and the MRI_200 model (in beige). The ground truth tumor area and Dice scores are also shown for each case. (The figure is best viewed in color in a digital version, which allows for zooming in).

A comparison of the evaluation metrics Dice score, recall, precision, and FNR obtained on all 14,107 2D slices from the 29 iUS annotated patients for the nine models trained with different tumor area cut-off values, are represented by box plots in Figure 2. Dice and recall increased significantly for increasing cut-off ($p<10^{-5}$), and FNR decreased significantly ($p<10^{-9}$), whereas precision showed no significant trend (p=0.071). Examples of predicted segmentation masks from the MRI_0, MRI_35, and MRI_200 model, and the ground truth are shown in Figure 3, represented with the same colors as in the box plots. The Dice scores and ground truth tumor areas are also shown for each case.

The average Dice score and standard deviation for the entire and different tumor area ranges of the test set for the models are shown in the upper part of Table 1. In addition, it shows the percentage of positive predictions in the entire test set and the number of training samples N for each model. Statistical significance and effect size of the models compared to the MRI_0 model are also shown. The linear regression showed that the percentage of positive prediction (PP%) increased significantly with increasing tumor area cut-off ($p<10^{-16}$).

### 3.2. Comparison Among MRI Labels, iUS Labels, and Inter-Observer Variability

**Figure 4:**
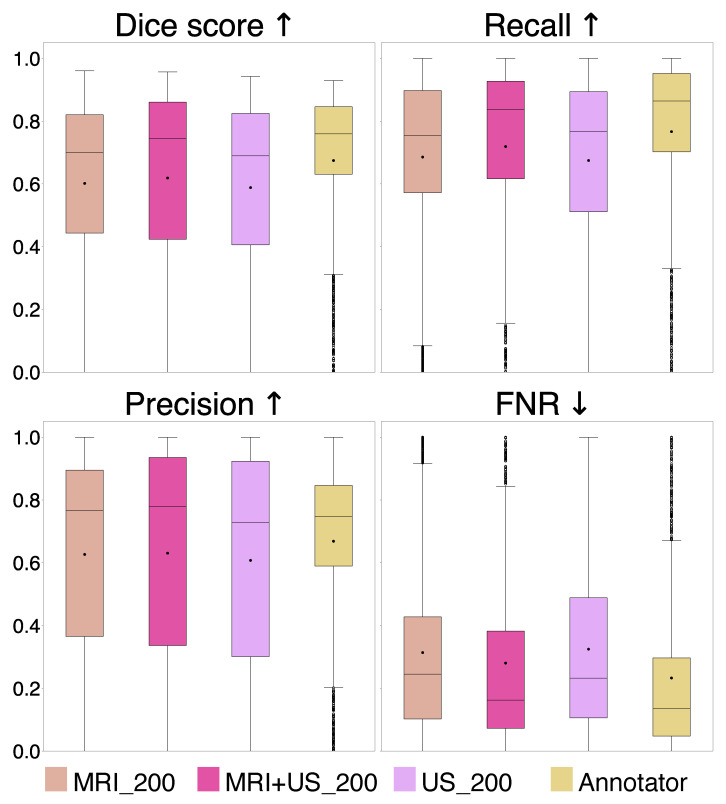
Comparison of Dice score, recall, precision, and false negative rate (FNR) for the MRI_200, MRI+US_200, and US_200 models, and for the Annotator. The arrows indicate which value is preferred.

A comparison of the evaluation metrics Dice, recall, precision, and FNR, are represented by box plots in Figure 4, for the MRI_200 model, MRI+US_200 model, US_200 model, and the Annotator.

**Figure 5:**
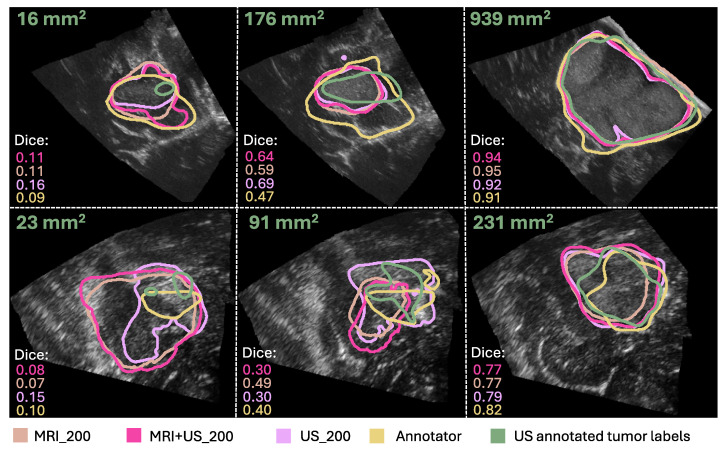
Comparison of the segmentation masks of the ground truth (in green), the Annotator (in yellow), the MRI_200 model (in beige), the MRI+US_200 model (in pink), and the US_200 model (in purple). The ground truth tumor area and Dice scores are also shown for each case. (The figure is best viewed in color in a digital version, which allows for zooming in.).

Examples of segmentation masks from the models and the Annotator are shown in Figure 5, represented with the same colors as in the box plots, and the ground truth for test slices with different tumor areas.

The average Dice score and standard deviation for different tumor area ranges of the test set and for the entire test set are shown in the lower part of Table 1, with statistical results shown in Table 2, for Experiment 2. In addition, the percentage of positive predicted cases of the test set and the number of training samples (N) for each model are shown in Table 1.

## 4. Discussion

The purpose of this study was to investigate whether MRI tumor annotations can successfully be used as a replacement for iUS tumor annotations, for training an automatic brain tumor segmentation model for iUS images.

In Experiment 1, we found that the models’ performance improves by increasing tumor area cut-off values in the training set as evidenced by a significant increase in Dice score, recall, and PP, and a significant decrease in FNR (p<0.0064, Bonferroni) compared to using all tumor areas (MRI_0), shown in Figure 2 and Table 1. The precision remains unchanged (p>0.0064, Bonferroni), suggesting that the ability to detect tumor pixels improves with larger tumor area cut-off values. This is illustrated in Figure 3, showing similar segmentation masks for all models except the MRI_0 and MRI_35 models where the tumor is missed in some cases. In addition, we found that a tumor area cut-off value around 200 mm^2^ seems to provide the best and most stable results. This is indicated by improvements in Dice score stagnates with a tumor cut-off value at 200 mm^2^, regardless of tumor area in the test set, and by the MRI_200 model having the highest effect size, as shown in Figure 2 and Table 1. This value was, therefore, used in Experiment 2.

A possible explanation for the improvement in model performance when excluding the smallest tumors from the training set could be that the inaccuracies in the image registration have a larger impact on the smaller tumors, and including these might add more noise to the training data rather than valuable information. This illustrates that higher quality over quantity in the training data might be a good compromise in this case, as including the smaller tumors increases the training sample size, but decreases the performance. In addition, the segmentation task itself could be more challenging for smaller tumors because the slices with smaller tumor areas are often from the tumor border, which is infiltrating healthy tissue, and could thus give a poorer contrast in the ultrasound images than the larger tumors areas. This can be seen in Figure 3. Additionally, segmentation of small structures is a well-known challenge in medical image segmentation because the amount of tumor pixels is low compared to background pixels, giving a high class-imbalance. Furthermore, the Dice score, which is used in the loss function of nnU-Net combined with cross-entropy, is highly sensitive to errors in small structures [25,29], and including small structures in the training could be disadvantageous for the network update.

From the results in Experiment 2, we found indistinguishable performances from models trained with different label origins, evidenced by similar scores and no significant differences (*p* >0.0085, Bonferroni) and very small effect sizes (d∼0.01), as shown in Table 1 and Table 2, Figure 4. The average Dice scores were 0.62±0.31, 0.59±0.28, and 0.58±0.32 for the MRI+US_200, US_200, and MRI_200 model, respectively. This indicates that MRI tumor annotation can be used as labels for ultrasound images. As a result, clinicians can save valuable time by reducing time spent on manually annotating ultrasound images to obtain a larger dataset for developing deep learning models for ultrasound. This would speed up the process of obtaining real-time models that could help neurosurgeons interpret ultrasound images during surgery, which could be very useful to assess remaining tumor tissue. However, the results did not improve even though the MRI+US_200 model was trained on a dataset approximately seven times larger than the one used for training the US_200 models. Table 1 and Figure 5 show that the models in this experiment also have poor results on small tumors, which could indicate that small tumors limit further improvements.

In addition, the results showed that the Annotator obtained an average Dice score of 0.67±0.25 on the test set, and 0.20±0.23 on the small tumors. These low scores illustrate how challenging the segmentation task is, even for expert neurosurgeons, and that there is a high inter-observer variability. In addition, it shows that the small tumors also obtain very poor Dice scores for the expert neurosurgeon, which are illustrated in Figure 5. However, the Dice score is not a good evaluation metric for small structures because it is highly sensitive to minor changes in small structures [29]. Other evaluation metrics should thus be explored to better evaluate the results using smaller tumors. For instance, boundary based metrics, such as normalized surface distance or boundary intersection over union, could be explored instead of overlap-based metrics, such as the Dice score [30].

Compared to the deep learning models, the Annotator achieved superior scores for all metrics on the entire test set, although no statistically significant differences were found (*p* >0.0085, Bonferroni), and the effect sizes were small (d∼0.01). However, the statistical analysis was limited by the size of the test set. Although the number of 2D slices was large, the small patient sample (*n* = 6) makes the performance highly sensitive to the test patients. A larger test set with diverse tumor types, annotated by multiple experts, would be in favor of a more robust statistical analysis, and better evaluation of the models. Nevertheless, the Dice scores obtained on the larger tumors (>200 mm^2^) were similar to the scores achieved by the deep learning models, indicating expert-level performance for larger tumors.

Compared to previous work, Qayyum et al. [8] used a self-supervised learning based 3DResUNet model using the RESECT dataset, where the encoder was trained using two augmented views of the same 3D patch. Their approach obtained an average Dice score of 0.57 on the same test set in 3D, compared to 0.62 for the MRI+US_200 model in 2D. Even though this is not directly comparable, it indicates that our approach achieves comparable or better results, and supports the usage of MRI tumor annotations. However, it could be interesting to combine their self-supervised approach to our method to see if that improves the results. The patient-specific approach of Dorent et al. [11], achieved better results with a median Dice score of 0.84–0.87, compared to a median Dice score of 0.74 for the MRI+US_200 model. However, these models were fine tuned to each patient in the training dataset, requiring time and resources that make a clinical adaption of the models challenging. Nevertheless, they used synthetic ultrasound-images generated from MRI which also could be interesting to test to increase the size of the dataset.

Despite promising results using MRI tumor annotations as a substitute for iUS annotations, the task of intra-operative brain tumor segmentation remains challenging. Although the models achieved promising performance on larger tumors, more thorough testing and validation is needed before clinical use. For instance, the cross-domain testing should be carried out using a larger test set of ultrasound images of various tumor types from different users, hospitals, and scanners where the image quality could be different to make sure it works across domains. Ultrasound images from different hospitals and scanners should also be included in the training set to make the model more robust for variations in ultrasound images. In addition, the detection of small structures remains a challenge. It is crucial for extending the model to segmentation of tumor tissue during and after resection, and should be the focus of future work. An overall limitation of this study is the small number of patients. Tumor annotations in both pre- and post-operative MRI scans from patients who have undergone brain tumor resection surgery, with corresponding iUS images, can be included to increase the number of smaller tumors in the training set. Different methods for improving the registration of the MRI labels to the iUS tumor, such as affine or non-linear registration methods or manual adjustment, could be explored. Additionally, boundary based evaluation metrics and loss functions tailored for small structures, other U-Net architectures, a fundamentally different deep learning network like a vision transformer network, or foundation models like USFM [31], could be tested to improve the results. Other strategies than semantic segmentation could also be investigated, such as bounding boxes proposed by Weld et al. [32].

## 5. Conclusions

Although the detection of small tumors remains a challenge, our study showed that MRI tumor annotations can successfully be used as substitute labels for unannotated iUS images to train an automatic brain tumor segmentation model for 2D iUS images.

## Figures and Tables

**Table 2:** *p*-values from a pairwise comparison between the Dice scores on the test set for the MRI_200, MRI+US_200, US_200 models, and the Annotator are shown in the upper right part of the table, where *p* >0.0085 (Bonferroni) indicates statistical significance. The effect size of the differences are quantified by Cohen’s *d*, with d∼0.01 indicating very small effect size and d∼0.2 indicating small effect sizes, are shown on the bottom left side.

	MRI_200	MRI+US_200	US_200	Annotator
MRI_200	-	0.062	0.823	0.247
MRI+US_200	0.036	-	0.085	0.420
US_200	0.005	0.036	-	0.235
Annotator	0.047	0.030	0.047	-

## Data Availability

The original data presented in the study are openly available in Norstore at https://archive.norstore.no/pages/public/datasetDetail.jsf?id=10.11582/2017.00004 (accessed on 1 October 2024), and The Cancer Imaging Archive at https://doi.org/10.7937/3RAG-D070.

## References

[1] Delgado-López P.D., Corrales-García E.M., Martino J., Lastra-Aras E., Dueñas-Polo M.T. (2017). Diffuse low-grade glioma: A review on the new molecular classification, natural history and current management strategies. Clin. Transl. Oncol..

[2] Kheirollahi M., Dashti S., Khalaj Z., Nazemroaia F., Mahzouni P. (2015). Brain tumors: Special characters for research and banking. Adv. Biomed. Res..

[3] Sanai N., Berger M.S. (2008). GLIOMA EXTENT OF RESECTION AND ITS IMPACT ON PATIENT OUTCOME. Neurosurgery.

[4] Sastry R., Bi W.L., Pieper S., Frisken S., Kapur T., Wells W., Golby A.J. (2017). Applications of Ultrasound in the Resection of Brain Tumors. J. Neuroimaging.

[5] Cepeda S., García-García S., Arrese I., Sarabia R. (2024). Non-navigated 2D intraoperative ultrasound: An unsophisticated surgical tool to achieve high standards of care in glioma surgery. J. Neuro-Oncol..

[6] Carton F.X. (2021). Image Segmentation and Registration Using Machine Learning for Brain Shift Compensation in Image-Guided Neurosurgery. Ph.D. Thesis.

[7] Xiao Y., Yang G., Song S. (2023). Lesion Segmentation in Surgical and Diagnostic Applications: MICCAI 2022 Challenges, CuRIOUS 2022, KiPA 2022 and MELA 2022, Held in Conjunction with MICCAI 2022, Singapore, 18–22 September 2022, Proceedings.

[8] Qayyum A., Mazher M., Niederer S., Razzak I., Xiao Y., Yang G., Song S. (2023). Segmentation of Intra-operative Ultrasound Using Self-supervised Learning Based 3D-ResUnet Model with Deep Supervision. Lesion Segmentation in Surgical and Diagnostic Applications. MICCAI 2022 Challenges, CuRIOUS 2022, KiPA 2022 and MELA 2022, Held in Conjunction with MICCAI 2022, Singapore, 18–22 September 2022, Proceedings.

[9] Xiao Y., Fortin M., Unsgård G., Rivaz H., Reinertsen I. (2017). REtroSpective Evaluation of Cerebral Tumors (RESECT): A clinical database of pre-operative MRI and intra-operative ultrasound in low-grade glioma surgeries. Med. Phys..

[10] Behboodi B., Carton F.X., Chabanas M., De Ribaupierre S., Solheim O., Munkvold B.K.R., Rivaz H., Xiao Y., Reinertsen I. (2022). RESECT-SEG: Open access annotations of intra-operative brain tumor ultrasound images. arXiv.

[11] Dorent R., Torio E., Haouchine N., Galvin C., Frisken S., Golby A., Kapur T., Wells W. (2024). Patient-Specific Real-Time Segmentation in Trackerless Brain Ultrasound. arXiv.

[12] Juvekar P., Dorent R., Kogl F., Torio E., Barr C., Rigolo L., Galvin C., Jowkar N., Kazi A., Haouchine N. (2023). ReMIND: The Brain Resection Multimodal Imaging Database. medRxiv.

[13] Bouget D., Alsinan D., Gaitan V., Helland R.H., Pedersen A., Solheim O., Reinertsen I. (2023). Raidionics: An open software for pre- and postoperative central nervous system tumor segmentation and standardized reporting. Sci. Rep..

[14] (2022). CuRIOUS 2022. Brain Shift with Intraoperative Ultrasound—Segmentation Tasks—Grand Challenge.

[15] Gronningsaeter A., Kleven A., Ommedal S., Aarseth T.E., Lie T., Lindseth F., Langø T., Unsgård G. (2000). SonoWand, an ultrasound-based neuronavigation system. Neurosurgery.

[16] Askeland C., Solberg O.V., Bakeng J.B.L., Reinertsen I., Tangen G.A., Hofstad E.F., Iversen D.H., Våpenstad C., Selbekk T., Langø T. (2016). CustusX: An open-source research platform for image-guided therapy. Int. J. Comput. Assist. Radiol. Surg..

[17] Cancer Registry of Norway (2023). Norwegian Registry of Brain and Spinal Cord Tumours. https://www.kreftregisteret.no/en/The-Registries/clinical-registries/Quality-registry-for-brain-tumours/.

[18] Bouget D., Pedersen A., Jakola A.S., Kavouridis V., Emblem K.E., Eijgelaar R.S., Kommers I., Ardon H., Barkhof F., Bello L. (2022). Preoperative Brain Tumor Imaging: Models and Software for Segmentation and Standardized Reporting. Front. Neurol..

[19] Majewska P., Holden Helland R., Ferles A., Pedersen A., Kommers I., Ardon H., Barkhof F., Bello L., Berger M.S., Dunås T. (2025). Prognostic value of manual versus automatic methods for assessing extents of resection and residual tumor volume in glioblastoma. J. Neurosurg..

[20] Kommers I., Bouget D., Pedersen A., Eijgelaar R.S., Ardon H., Barkhof F., Bello L., Berger M.S., Conti Nibali M., Furtner J. (2021). Glioblastoma Surgery Imaging—Reporting and Data System: Standardized Reporting of Tumor Volume, Location, and Resectability Based on Automated Segmentations. Cancers.

[21] ImFusion (2018). ImFusion—ImFusion Suite. https://www.imfusion.com/products/imfusion-suite.

[22] Wein W., Brunke S., Khamene A., Callstrom M.R., Navab N. (2008). Automatic CT-ultrasound registration for diagnostic imaging and image-guided intervention. Med Image Anal..

[23] Wein W., Ladikos A., Fuerst B., Shah A., Sharma K., Navab N., Mori K., Sakuma I., Sato Y., Barillot C., Navab N. (2013). Global Registration of Ultrasound to MRI Using the LC2 Metric for Enabling Neurosurgical Guidance. Proceedings of the Medical Image Computing and Computer-Assisted Intervention—MICCAI.

[24] Fuerst B., Wein W., Müller M., Navab N. (2014). Automatic ultrasound–MRI registration for neurosurgery using the 2D and 3D LC2 Metric. Med. Image Anal..

[25] Isensee F., Jäger P.F., Full P.M., Vollmuth P., Maier-Hein K.H., Crimi A., Bakas S. (2021). nnU-Net for Brain Tumor Segmentation. Brainlesion: Glioma, Multiple Sclerosis, Stroke and Traumatic Brain Injuries, Proceedings of the 7th International Workshop, BrainLes 2021, Virtual Event, 27 September 2021.

[26] Fedorov A., Beichel R., Kalpathy-Cramer J., Finet J., Fillion-Robin J.C., Pujol S., Bauer C., Jennings D., Fennessy F., Sonka M. (2012). 3D Slicer as an image computing platform for the Quantitative Imaging Network. Magn. Reson. Imaging.

[27] Cohen J. (2013). Statistical Power Analysis for the Behavioral Sciences.

[28] Sawilowsky S. (2009). New Effect Size Rules of Thumb. J. Mod. Appl. Stat. Methods.

[29] Reinke A., Tizabi M.D., Sudre C.H., Eisenmann M., Rädsch T., Baumgartner M., Acion L., Antonelli M., Arbel T., Bakas S. (2023). Common Limitations of Image Processing Metrics: A Picture Story. arXiv.

[30] Maier-Hein L., Reinke A., Godau P., Tizabi M.D., Buettner F., Christodoulou E., Glocker B., Isensee F., Kleesiek J., Kozubek M. (2022). Metrics reloaded: Recommendations for image analysis validation. arXiv.

[31] Jiao J., Zhou J., Li X., Xia M., Huang Y., Huang L., Wang N., Zhang X., Zhou S., Wang Y. (2024). USFM: A universal ultrasound foundation model generalized to tasks and organs towards label efficient image analysis. Med. Image Anal..

[32] Weld A., Dixon L., Anichini G., Patel N., Nimer A., Dyck M., O’Neill K., Lim A., Giannarou S., Camp S. (2024). Challenges with segmenting intraoperative ultrasound for brain tumours. Acta Neurochir..

